# Microbial Ecology of Four Coral Atolls in the Northern Line Islands

**DOI:** 10.1371/journal.pone.0001584

**Published:** 2008-02-27

**Authors:** Elizabeth A. Dinsdale, Olga Pantos, Steven Smriga, Robert A. Edwards, Florent Angly, Linda Wegley, Mark Hatay, Dana Hall, Elysa Brown, Matthew Haynes, Lutz Krause, Enric Sala, Stuart A. Sandin, Rebecca Vega Thurber, Bette L. Willis, Farooq Azam, Nancy Knowlton, Forest Rohwer

**Affiliations:** 1 Department of Biology, San Diego State University, San Diego, California, United States of America; 2 School of Biological Sciences, Flinders University, Adelaide, South Australia, Australia; 3 Center for Marine Biodiversity and Conservation, Scripps Institution of Oceanography, University of California San Diego, La Jolla, California, United States of America; 4 Center for Microbial Sciences, San Diego State University, San Diego, California, United States of America; 5 Fellowship for Interpretation of Genomes, Burr Ridge, Illinois, United States of America; 6 Center for Biotechnology (CeBiTec), Bielefeld University, Bielefeld, Germany; 7 Australian Research Council (ARC) Centre of Excellence for Coral Reef Studies, School of Marine and Tropical Biology, James Cook University, Townsville, Queensland, Australia; Centre for DNA Fingerprinting and Diagnostics, India

## Abstract

Microbes are key players in both healthy and degraded coral reefs. A combination of metagenomics, microscopy, culturing, and water chemistry were used to characterize microbial communities on four coral atolls in the Northern Line Islands, central Pacific. Kingman, a small uninhabited atoll which lies most northerly in the chain, had microbial and water chemistry characteristic of an open ocean ecosystem. On this atoll the microbial community was equally divided between autotrophs (mostly *Prochlorococcus* spp.) and heterotrophs. In contrast, Kiritimati, a large and populated (∼5500 people) atoll, which is most southerly in the chain, had microbial and water chemistry characteristic of a near-shore environment. On Kiritimati, there were 10 times more microbial cells and virus-like particles in the water column and these microbes were dominated by heterotrophs, including a large percentage of potential pathogens. Culturable *Vibrio*s were common only on Kiritimati. The benthic community on Kiritimati had the highest prevalence of coral disease and lowest coral cover. The middle atolls, Palmyra and Tabuaeran, had intermediate densities of microbes and viruses and higher percentages of autotrophic microbes than either Kingman or Kiritimati. The differences in microbial communities across atolls could reflect variation in 1) oceaonographic and/or hydrographic conditions or 2) human impacts associated with land-use and fishing. The fact that historically Kingman and Kiritimati did not differ strongly in their fish or benthic communities (both had large numbers of sharks and high coral cover) suggest an anthropogenic component in the differences in the microbial communities. Kingman is one of the world's most pristine coral reefs, and this dataset should serve as a baseline for future studies of coral reef microbes. Obtaining the microbial data set, from atolls is particularly important given the association of microbes in the ongoing degradation of coral reef ecosystems worldwide.

## Introduction

The roles of microbes, both Bacteria and Archaea, and viruses on coral reefs are just starting to be elucidated. Most studies concern microbes in the water column, although actual densities are much higher in the benthos [1]. Microbes may play an important role in the nutrition of reef organisms. For example, the number of microbes in the water column declines from the windward to leeward (forereef to backreef) areas of coral reefs [2], suggesting ingestion by coral reef organisms [3]–[5]. Similarly, decreasing densities of bacteria have also been documented within the vertical structure of a coral reef, with the over-lying water column containing approximately 4.5 times the amount of bacteria compared with the water within crevices of the coral reef structure [6].

Our ability to understand these microbes has increased greatly with the development of molecular and genomic approaches that provide a far more accurate picture of community composition and activities. In the marine environment molecular techniques have identified new organisms and new metabolic processes [7]. For coral reefs, molecular techniques, such as 16S rDNA analysis has identified that microbial communities associated with corals are diverse and develop both species specific [8], and generalist associations [9]. These molecular techniques have also revealed the etiological agents of diseases of coral reef organisms, such as, corals [10] and sponges [11], [12]. In some cases the etiological agents are not specific to corals, but infect multiple and distinctive marine organisms [13], leading to difficulties in identify causative agents of the increasing number coral diseases that are described [14]. The lack of identified pathogens suggests opportunistic bacterial infections or hard-to-culture pathogens (e.g., viruses) are important mediators of coral disease. Because of these difficulties, metagenomics, which allows the entire genome of all the micro-organisms within an environment to be sampled rapidly [15], may be required to describe microbial associations on coral reefs and how they change with environmental fluctuations and anthropogenic activities. Metagenomic studies are not restricted by targeting single gene regions, but provide information on all genomic regions, enabling both taxonomic descriptions and potential metabolic functions of the micro-organisms within an environment to be described [16]. For coral reefs, a comprehensive evaluation of the microbial and viral community may be particularly important because coral diseases are an increasing factor in the global collapse of reef ecosystems [14], [17].

In this study, the coral reef microbial communities associated with four coral atolls in the Northern Line Islands (central Pacific) were surveyed. These atolls are of interest because although they are relatively close to each other (750 km), they span an array of oceanographic conditions and are variably impacted by human activities. One of the atolls is pristine with respect to local anthropogenic effects, and thus provides an important microbial baseline against which other reefs can be compared. Microbial and viral abundances were complemented with metagenomic analyses of these communities. Coral cover and disease prevalence were also measured to determine if there were correlations between microbial communities and coral health. A companion study complements the microbial data by measuring functional changes of the fish and benthic communities [18].

## Materials and Methods

### Study sites

**Figure 1:**
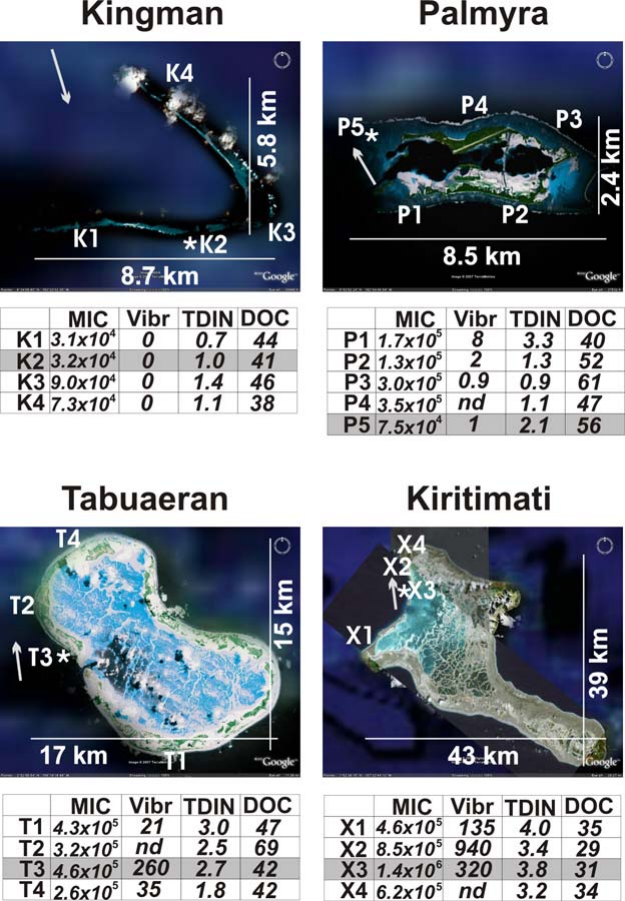
Maps of the sites surveyed on the four Northern Line Island atolls. The locations for the water chemistry, microbe/viral direct counts, and *Vibrio* spp. culturing are indicated with the first letter of the atoll name (X for Kiritimati sites) and sequentially numbered. The sites for the metagenomes are labeled with an *. Coral cover, fish counts, and other macro-organism data were sampled at all of these sites, as well as additional sites [18]. The prevailing current is shown as a grey arrow. MIC = number of microbes per ml; Vibr = number of culturable *Vibrio* spp. on TCBS plates per ml; DOC = dissolved organic carbon in µM; TDIN = total dissolved inorganic nitrogen in µM (nitrite and nitrate, and ammonium). Maps were taken from Google Earth.

Surveys were conducted on four atolls in the Northern Line Islands in the central Pacific (Figure 1; [18]). The atolls are separated by ∼750 km and span a gradient in oceanic productivity and climate. The level of rainfall declines from north to south [19], whereas oceanic productivity declines from south to north.

Two atolls, Kingman and Palmyra, are part of the US national refuge system and have little or no local anthropogenic impacts. The most northern atoll, Kingman, is uninhabited and has only a few emergent sand bars (<0.1 km^2^), and the reef crest surrounds a relatively large lagoon (60 km^2^). As such, it is expected to have a high flushing rate and no terrestrial influence of any kind (e.g., human sewage, bird guano, agricultural runoff). Palmyra has ∼7 km^2^ of land and a relatively small lagoon (15 km^2^) that was extensively remodeled during World War II. There are only ∼20 people on Palmyra at any one time, and sewage is treated and contained. Seabirds are numerous and a potential source of nitrogen, but guano was never mined on Palmyra because it does not accumulate in significant quantities. Ammunition dumps are also potential sources of nitrogen compounds. There are a number of small wrecks and former military structures, which may increase local levels of iron. Shark finning occurred on Palmyra in the past.

The remaining two atolls, Tabuaeran and Kiritimati, are inhabited and part of the Republic of Kiribati. Tabuaeran has 34 km^2^ of land and a 110 km^2^ lagoon; a natural passage on the west side of the island was widened by blasting of coral heads by the British, in the 1890s. There are ∼2500 people and a large wreck just north of the lagoon passage. All that visually remains of this wreck are a number of large boilers. Kiritimati has 390 km^2^ of land and 324 km^2^ of lagoon, and ∼5500 people. Human sewage is untreated on both Tabuaeran and Kiritimati and there was no evidence of any traditional management of sewage. There are some septic tanks associated with the hotel and larger buildings on Kiritimati; many of these leak, and combined with the untreated sewage, there are a number sanitation problems associated with the island's water supply. Recently installed composting toilets are often not used because of local customs and beliefs (http://www.unep.or.jp/ietc/publications/techpublications/techpub-15/3-8IlandPacific/8-11-2_1.asp). There have been several agriculture initiatives on Tabuaeran and Kiritimati, including seaweed and coconuts. Guano was mined on Kiritimati in the 1850's and 1860's, but it is not known how much mining actually took place; mining stopped in 1866 because it was unproductive. Atomic bomb testing was conducted on Kiritimati between 1957 and 1962 by both the British and American military. Kiritimati has a series of lagoons, a large main one and then many smaller lagoons that are both connected and unconnected to the main lagoon. The highest fluctuations in physio-chemical properties occurs in the unconnected lagoons [20], [21], however the main lagoon has salinities and pH similar to seawater. Dissolved oxygen levels were low in the main lagoon and found to increase only in close proximity to settlements, suggesting eutrophication of small areas [20]. Microbial activity in the lagoon sediments was high, but similar to levels in other Pacific lagoons [21].

### Survey overview

Microbial communities were surveyed in the coral reef waters on each of the four atolls at 10–12 m depth (<400 m from shore), between August 4^th^ and September 6^th^ 2005. We used the following approaches: 1) Quantification of Bacteria and Archaea (microbes), virus-like particles (VLPs), and protists using direct counts on water collected from above the reef substratum, 2) Abundances of culturable *Vibrio* spp. determined by counting colony forming units (cfu) on thiosulfate citrate bile sucrose plates (TCBS), and 3) Taxonomical and metabolic potential of the microbial and viral communities using metagenomic analyses. In addition, we characterized the coral community (percent cover, disease prevalence) and the water chemistry [concentrations of total dissolved inorganic nitrogen compounds (TDIN: ammonium, nitrate, nitrite), phosphate, and dissolved organic carbon (DOC)].

At each atoll we used the same sampling strategy (one site for metagenomic analyses, four to five sites for other microbial and water chemistry samples, 10–12 sites to characterize the benthic community, ∼30 sites (separated by 2 km) to characterize the fish community). The general sampling scheme was centered at the leeward side and worked out in both directions around the island (Figure 1, see Sandin et al [18]; further details below). Because of the differing rates at which the fish, benthic, and microbial surveys could be conducted, not all groups were characterized at each site. Thus about 50% of total sites sampled for coral cover and other benthic properties were also microbiologically characterized.

For the metagenome samples, areas underneath the lagoon currents were targeted because: 1) It was expected that these areas would be the most likely to show signs of human disturbance, 2) Time and resources limited the survey to one microbial and one viral metagenome per atoll, and 3) This limited sampling meant that it was necessary to target an area of the reef that had similar hydrological characters, and lagoon currents are a relatively constant feature of coral reefs. The lagoons of Palmyra, Tabuaeran, and Kiritimati tend to flush in a northerly direction. The samples for the metagenomes were taken from benthic sites that are flushed with the lagoonal waters. In the case of Kingman, the water flows over the reef. The prevailing current, during the cruise was from the north to south (as determined with a float), so this metagenome sample was taken on one of the gaps on the southside of the atoll (Figure 1).

### Direct counts of Bacteria, Archaea and virus-like particles

The numbers of microbes (both Bacteria and Archaea) and virus-like particles (VLPs) in the water column were determined via direct counts using epifluorescent microscopy. Pre-washed diver-adapted polycarbonate Niskin bottles were used to sample the water at each site. Each Niskin bottle sampled 2 liters of seawater, which was used for the direct counts, culturing, and water chemistry. Four of the bottles were collected from the reef surface (∼10 m depth), two bottles were collected 25 cm above the benthos, and two bottles were collected 500 cm above the benthos. No statistical difference was found between counts taken at various depths (F*_32_* = 0.321, P = 1 for microbes and F*_32_* = 0.320, P = 1.0 for VLPs), therefore samples were analyzed at the site level. The counts were conducted on 2 or 8 ml of sea water (two concentrations were prepared to ensure that we obtained countable slides). The samples were fixed with electron microscopy-grade paraformaldehyde (4% final concentration) and stained with SYBR Gold (1× final concentration; formally Molecular Probes, Inc., now Invitrogen, Solana Beach, CA) and filtered onto 0.02 µm Anodisc filters (Whatman, Inc, Florham Park, NJ), mounted on glass slides and directly counted by epifluorescence microscopy. Cells and VLPs were counted (>200 per sample) in 10 fields selected at random. The microbes and VLPs counts were log transformed and compared using an unbalanced multivariate analysis of variance with sites nested within atolls. Normality and heterogeneity were tested using Kolmogorov-Smirnov and Levene tests, respectively. Atoll pairings were tested using a Wilcoxon one-sided analysis.

### Enumeration of protests

The protist samples were counted from 100 ml of seawater taken from each of the Niskin bottles, fixed with Lugol's solution and formalin, de-stained with sodium thiosulfate, filtered onto 0.6 µm polycarbonate membranes (Millipore, Billerica, MA) and stored at −20°C. Filters were stained for three minutes with 400 µl DAPI (1 µg ml^−1^; Sigma), rinsed with deionized water and mounted onto glass slides using Vectashield (Vector Laboratories, Inc.; Burlingame, CA) [22]. Filters were observed via epifluorescence microscopy with a BX51 microscope (Olympus America, Inc) and protist abundances were determined based on average counts of ∼20 fields per filter. Pigmented and non-pigmented protists were differentiated using the TRITC band excitation filter set (excitation ∼550 nm; emission ∼600 nm). Pigmented protists were classified as autotrophic-mixotrophic and non-pigmented protists as strictly heterotrophic. Protist count data were normally distributed and homogenous and therefore were not transformed (tested as described above). An unbalanced ANOVA with sites nested within atolls was used to analyze the data, and Tukey's post-hoc test was used to identify the difference between atolls.

### Microbial and viral metagenomes

A sample of approximately 150 l of seawater was collected at one site per atoll (Figure 1). The water for the metagenomes was collected from below the boundary layer (in crevices and against the benthos) to avoid confounding problems with the water column. The sampling was conducted at the same time of day to help minimize diurnal effects. The water was collected from over ∼20 m^2^ of reef using a modified bilge pump connected to low density polyethylene (LDPE) collapsible bags (19 l; Cole-Parmer, Vernon Hills, IL; Figure S1). The containers were transported to the surface and the research vessel within two hours of collection, thereby reducing potential *in situ* community changes. To remove potential sources of DNA contamination, containers, bilge pumps, and tubing were washed once with 10% bleach, three times with freshwater, and once with 100 kDa filtered seawater prior to sampling.

Two size fractions were prepared for the metagenomic analysis from the seawater samples: 1) A large fraction containing mostly microbes, some small eukaryotes (such as dinoflagellates and protists), and a few VLPs, and 2) a small fraction containing mostly VLPs and some small microbes. To obtain these fractions the seawater was processed through a series of filters. The large eukaryotes were removed by filtering the entire sample through 100 µm Nitex, into a barrel lined with a clean, high-density polyethylene bag. The filtrate was then concentrated to ∼500 ml on a 100 kDa tangential flow filter (TFF), which captured the unicellular eukaryotes, microbes and VLPs (i.e., the water was removed). During the filtration, pressures were kept below 0.6 bar (10 psi) to ensure that the viruses were not destroyed. The concentrated sample was then passed through 0.45 µm Sterivex filters (Millipore, Inc) using a 50 ml syringe. In this step, the large metagenomic fraction consisting of microbial cells was caught on the filter (microbiomes) and the filtrate was the small metagenomic fraction (viromes). All filtrations were performed on the research vessel, and the samples were stored for further processing in the laboratory at SDSU. The Sterivex filters were frozen at −80°C. The 0.45 µm filtrates (i.e., the virome) were extracted with chloroform to kill any residual cells (10% vol:vol; most viruses are resistant to chloroform) and stored at 4°C.

The DNA for the microbiomes was isolated from the Sterivex filters by removing the filter membranes and performing DNA extractions using a bead-beating protocol (MoBio, Carlsbad CA). The DNA obtained was amplified with Genomiphi (GE Healthcare Life Sciences, Inc, Piscataway, NJ) in six to eight 18-hour reactions [23]–[28]. The reactions were pooled and purified using silica columns (Qiagen Inc, Valencia, CA). The DNA was then precipitated with ethanol and re-suspended in water at a concentration of approximately 300 ng µl^−1^.

The viruses in the small metagenomic fractions (i.e., 0.45 µm filtrate treated with chloroform) were purified using cesium chloride (CsCl) step gradients to remove free DNA and any cellular material [29], [30]. Viral DNA was isolated using CTAB/phenol:chloroform extractions and amplified in six to eight 18-hour Genomiphi reactions. These reactions were pooled and purified using silica columns (Qiagen Inc, Valencia, CA). The DNA was then precipitated with ethanol and re-suspended in water at a concentration of approximately 300 ng µl^−1^.

Both the virome and microbiome DNAs were sequenced at 454 Life Sciences (Branford, CT) using their parallel pyrosequencing approach.

### Initial bioinformatics on metagenomes

The DNA sequences generated by 454 Life Sciences, Inc, were analyzed *without* assembly. This approach simplifies the statistical analysis and avoids problems with chimera assemblies. Thus, these sequences represent environmental gene tags [31]–short fragments of genes that are found in the different samples. To characterize these sequences, several independent approaches were taken. In the first approach, the sequences were compared to the extant sequence libraries using the Basic Local Alignment Search Tool (BLAST) algorithm. Several boutique databases were used for rapid comparisons and categorization of the DNA sequences. All sequences were compared to the 16S ribosomal DNA database (version 9) available from http://rdp.cme.msu.edu/; the Phage Genome Database and the viral genome database http://phage.sdsu.edu/phage; the European Ribosomal DNA database (http://www.psb.ugent.be/rRNA/); and the ACLAME (A Classification of Genetic Mobile Elements) database (http://aclame.ulb.ac.be/) [32]–[35]. Results from these analyses are all available at the Line Islands section of our website http://scums.sdsu.edu. Sequences were also compared to the SEED platform [36], [37] using the BLASTX algorithm [38]. This database contains the protein sequences from all the available complete and draft genomes. The comparisons were run on the Life Sciences Gateway to the Teragrid (Judson, Edwards, Papka, and Stevens, in preparation). The number of sequences obtained from the 454 pyrosequencing and the number that showed significant similarities are provided in Supplementary Table S1.

The metagenomic sequence fragments with significant similarities (E-value≥0.01) to the SEED platform (http://metagenomics.theseed.org/) were assigned functions based on their closest similarity. This approach allows rapid assessment of the metabolic potential of the sample and provide reliable taxonomic and functional assessments [29], [30], [39].

### Taxonomy and guild assignments

Taxonomical assignments of uncultured microbes are routinely based on sequence data. In the case of microbial metagenomes, this is done in two ways: 1) closest hits to 16S rDNAs, and 2) closest hits to any sequence from a known organism. In the former case, it is relatively easy to just compare the metagenome results to those obtained from traditional 16S rDNA surveys. Using this approach, it has been shown that both qualitatively and quantitatively, the 16S rDNA genes in metagenomes are very similar to those found by 16S rDNA sequencing [39]. This is true regardless of whether the metagenome is made by cloning and Sanger sequencing or by the cloneless 454 pyrosequencing approach. In the latter case, the shorter sequences (∼100 bp) yield the same overall result as the longer Sanger sequences. Though less established than the 16S rDNA approach, taxonomical assignment of metagenomic sequence fragments based on their closest hits to any sequence from a known organism is accurate and informative, and much more metagenomic data are utilized in the analysis. Ground-truthing in this case has been done by a variety of comparisons to coding genes, as well as to cloned 16S rDNA libraries [39]. This approach has also been validated for 454 pyrosequencing data [40].

Both 16S rDNA cloning approaches and metagenomics suffer from the fact that the microbes are not in culture. Very closely related microbes can have quite different phenotypes based on just a few genes (e.g., exotoxins). Therefore, assignment of microbes to guilds based on uncultured data needs to be qualified. For this study we chose to assign the metagenomic fragments to autrotrophs, heterotrophs, and potential pathogens (see below for details). While there might be some overlap between these guilds, the results are very similar whether just the 16S rDNA assignments are considered or functional assignments are used. Although as noted above, small genetic differences can result in major guild differences, the methods were applied consistently across all the atolls so that bias associated with the method would be systematic in nature.

The “habitat” of each microbe with a genome in the SEED platform was manually curated from Bergey's Manual of Determinative Bacteriology [41]. For example, cyanobacteria were assigned to the “autotroph” guild and SAR11 was assigned to the “heterotroph” guild. The “potential pathogen” guild consisted of metagenomic sequence fragments most closely related to the human pathogenic genera *Staphylococcus, Vibrio,* and *Escherichia,* the fish pathogens belonging to *Aeromona*, and plant pathogens from the *Xylella* genera. A complete list of the organisms that we described as autotrophic, heterotrophic or potential pathogens is provided on the Line Islands section of our accompanying website at http://scums.sdsu.edu under the section titled “Bergey's listing”. This categorization was used to estimate the proportion of microbes that could be described as belonging to one of these three groups on each atoll.

### SEED-based assignments of metabolic potential

The metabolic potential of each sample was identified by examining the similarities between the metagenomic sequence fragments and genes in metabolic subsystems. A metabolic subsystem is a group of genes that together form a metabolic function or pathway. The complete list of genes that was similar to metabolic subsystems for microbiomes is provided on http://scums.sdsu.edu. Descriptions of each of the subsystems are available from http://www.theseed.org/. The frequency that each metabolic subsystem was found at each atoll was visualized using a novel interactive web interface which color-codes the frequency to which each subsystem is found in each metagenome sample (http://metagenomics.theseed.org/).

Two different methods were used to calculate the statistical significance of the presence and absence of different metabolic subsystems in each sample: 1) The XIPE-TOTEC subsampling method [37], and 2) the G-test (a maximum likelihood test), neither of which depend on a normal distribution of the data. The two approaches identified which metabolic subsystems were statistically over-represented between the atolls (results are provided at http://scums.sdsu.edu). The analyses of the subsystems and accompanying statistics were used to calculate the seven most abundant subsystems that were different between Kingman and Kiritimati. Relative representation of each subsystem was calculated as the number of similarities to a particular subsystem divided by the total number of similarities to any subsystem; using percentages allows comparisons between samples regardless of the number of sequences obtained from each pyrosequencing reaction.

### Bioinformatics of the viromes

The small fraction metagenomic libraries were compared to the known phage genomes in the phage database (v. 5; http://phage.sdsu.edu/phage). This database contains 510 phage genomes and was used to construct the latest version of the Phage Proteomic Tree [34], [42]. To compare the relative numbers of phage hosts at each atoll, the incidence of each phage host genome per atoll was counted. Counts were normalized for the number of sequences per metagenome, enabling direct comparisons between metagenomes. The microbe that the phage infects was then compared against the “Bergey's List” to describe the phage as either infecting an autotrophic, heterotrophic or potentially pathogenic bacteria identified as described above.

Two individual phage and prophage strains (*Escherichia coli* Φ CP4-6 prophage and *Prochlorococcus marinus* SSMP4 phage) were used to provide information on the spread of sequence similarities across individual genomes. The coverage maps were constructed by creating a database with the phage or prophage genomes of interest and comparing the metagenomes against them by using TBLASTX. Sequences with E-values≤0.0001 were binned into 1000 base pair windows along the reference genomes. The total number of sequence similarities within a window was calculated and divided by the total number of sequences for each metagenome, allowing comparisons between metagenomes.

### Pfam and GO term analysis

The metagenomic libraries were also analyzed using the Pfam database as a comparison to the SEED platform conclusions. To identify conserved protein domains in the metagenomes, all sequence fragments from a sample were translated into each of the six reading frames. To save computational time, translated sequences with an in-frame stop codon were excluded from further analysis. The remaining sequences were locally aligned to each Pfam family using the pHMM from the Pfam_fs database (version 20.0). To search the Pfam_fs database, hmmpfam was run on a computer cluster at the Center for Biotechnology (CeBiTec; Bielefeld University, Germany) [43]. The E-value cut-off was set to 0.01.

Conserved protein domains were categorized into functional groups according to the Gene Ontology (GO) [44]. For each identified domain, GO terms were obtained from its Pfam family description. A pair-wise comparison of all samples versus all samples was performed to identify overrepresented GO terms in a sample. To determine if a GO term was significantly overrepresented, the G_adj_-test was employed under the null hypothesis H_0_: there is no difference in the abundance of identified domains in the two samples to which the GO term was assigned. Values of G were adjusted by the Williams' correction factor. The significance level was set to P<0.05.

To predict the species composition of a sample, 454 pyrosequencing fragments that had a Pfam hit were labeled according to the longest common prefix shared by the most similar members of the Pfam family. The strategy for assignment was as follows: First, the sequence was compared to each member of the Pfam family with a pair-wise alignment using BLAST. Second, *E_min_* was assigned as the minimal E-value obtained in the BLAST comparisons. The taxa of the organisms of all Pfam family members that hit the sequence with an E-value<*E*_min_+5%·*E*_min_ were fetched from NCBI. Finally, the sequence was labeled with the longest common prefix of these taxa. For example, *Bacteria:Cyanobacteria* is the longest common prefix of the two taxa

- *Bacteria:Cyanobacteria:Prochlorales:Prochlorococcaceae:Prochlorococcus*
- *Bacteria:Cyanobacteria:Chroococcales:Synechococcus*

The taxonomic identification provided by this analysis was categorized according to the definitions in Bergey's manual, thus enabling a comparison of the metagenomic data using a separate database.

### Vibrio spp. Culturing

Bacteria were cultured using thiosulfate citrate bile sucrose (TCBS; Remel; Lenexa, KS USA) plates to provide an estimate of the number of culturable *Vibrio* spp. present on each atoll. Water was sub-sampled from the Niskin bottles and 0.1 ml was inoculated on eight plates for each site. To determine the numbers of *Vibrio* spp. associated with coral, mucus samples were obtained from six corals per site using specially developed “super-suckers”. This apparatus allows the mucus to be gathered from the coral colony while minimizing the entry of surrounding seawater. Once again, 0.1 ml of coral mucus was spread onto plates. *Vibrio* spp. colonies forming units were enumerated after 24 hours incubation at 28°C (ambient sea water temperature). Differences in *Vibrio* spp. concentrations per ml of seawater or mucus were compared across all four atolls simultaneously using a non-parametric ANOVA. To further assess differences in the number of culturable *Vibrio* spp. between atolls, pairwise comparisons for all possible pairs of atolls were conducted using non-parametric exact Wilcoxon tests, testing a one-sided alternative hypothesis (i.e., that one atoll in the pair had a higher concentration of *Vibrio* spp. than the other). The one-sided test was conducted for all atoll pairings. On Kingman no culturable *Vibrio* spp. were observed in either the seawater or coral mucus, so the TCBS plates were tested with isolates from Kiritimati. In all cases, the plates were able to support growth of *Vibrio* spp.

### Water chemistry

Dissolved organic carbon (DOC), total dissolved inorganic nitrogen (nitrate, nitrite and ammonium), and dissolved inorganic phosphate were determined for all Niskin bottles. For DOC analysis, the water samples were filtered through a pre-combusted Whatman GF/F glass fiber filter and collected in pre-combusted amber glass vials (Wheaton) with acid-washed Teflon lined lids. Samples were acidified (∼pH 2) with analytical grade 30% hydrochloric acid (Fluka) and stored at 4°C. DOC concentrations were analyzed by Expert Chemical Analysis (San Diego, CA) using the high-temperature combustion method and a O.I. Analytical Model 1010 TOC analyzer (Texas, USA). To ensure quality control, DOC consensus reference materials (CRM: DSW Lot 05-05 at 45-46 µM C; LCW Lot 12-01 at 2 µM C; supplied by Dr Wenhao Chen, University of Miami) were used, and the high-carbon standard was run every six samples. The methods described for the DOC analysis are the same as those prescribed by the Intergovernmental Oceanographic Commission for the collection of DOC samples. These protocols avoid many of the uncertainties associated with earlier research on DOC levels [45], [46].

For inorganic nutrient analysis, samples were filtered through 0.2 µm Nuclepore Track-Etch membrane filters (Whatman) into HDPE scintillation vials with cone-shaped plastic lined lids (Fisher Scientific), after rinsing both the bottles and lids three times with filtrate. Each sample consisted of 15ml of filtrate, which were then stored at −20°C until analyzed. Analysis of inorganic nutrient (nitrate, nitrite, ammonium and phosphate) concentrations was carried out by Marine Science Institute Analytical Lab at University of California at Santa Barbara (Santa Barbara, CA) using a QuikChem 8000 flow injection analyzer (Lachat Instruments, Wisconsin, USA). All chemical components were found not to vary between depths (DOC F_32_ = 0.872, P = 0.660; TDIN F_32_ = 0.968, P = 0.572; Phosphate F_32_ = 1.048, P = 0.442), and therefore, analyses were conducted on the site level. All water chemistry variables were normally distributed and did not show heterogeneity (tested using test described earlier). The differences between the concentrations of each chemical component on each atoll were compared using a MANOVA with sites nested within locations.

To determine natural isotopes of nitrogen, 1 liter of seawater was filtered through GF/F filters to collect particulate organic matter. The filters were folded in half, wrapped in foil, and stored at −20°C. The ^14^N/^15^N ratios were determined at the Marine Sciences Institute at UCSB (Santa Barbara, CA) using a Finnigan Delta Plus Advantage with a Costech Elemental Analyzer peripheral. Measurements conducted at Kingman and Kiritimati only.

### Respiration experiments

Micro-respiration experiments were used to estimate the “nutritional quality” of the waters from the four atolls. On each atoll, water collected from three sites only, was 0.2 µm filtered to remove microbes, and stored frozen at −80°C. Upon return to San Diego State University, the samples were thawed and inoculated with the same microbial community to determine whether the waters from one atoll would support more or less microbial activity. The microbial community was obtained by centrifuging 1 ml of water from a laboratory coral reef tank at ∼5,000×g in a microfuge. The supernatant was aspirated away, and the microbial pellet was resuspended into 1 ml of the previously thawed water from the different atolls (each sample run in triplicate). The mixture of the aquarium microbes and atoll water was placed in micro-respirometry chambers and the oxygen concentrations were measured using micro-oxygen sensor probes (Unisense, Aarhus C, Denmark). The microbes were grown for a total of four hours and oxygen concentrations (nmol O_2_ ml^−1^) were measured every minute. Negative controls were conducted by running the experiments without microbial inoculations. To calculate respiration rates, the slopes of the oxygen concentration curve between 75 to 175 minutes were determined. The timeframe was used because it produced the most consistent oxygen utilization levels (i.e., a linear slope after the lag phase). The number of cells in each chamber was determined by direct counts (described above) at the end of the experiment and the respiration rate per million microbes was calculated by dividing by the number of microbial cells. Conventional methods of measuring growth rates of microbes (e.g., using ^3^[H]-thymidine) were not performed because there was no access to a “rad van” on the cruise. Measurements of microbial activity using Br-dUTP incorporation were attempted, but failed because of technical problems. In the end, however, measuring the oxygen consumption by the same microbial community in the different water samples was probably more illustrative than comparing growth rates of different communities in different waters.

### Coral health

Surveys describing the health status of corals were conducted on two 2 m×20 m belt transects at 10–12 sites on each of the four atolls. These techniques for the assessment of coral health were developed by the Coral Disease working group of the Global Environmental Facility/World Bank coral reef targeted research program [14]. All coral colonies, both healthy and those showing signs of potential disease were counted. Most coral diseases do not have known microbial pathogens, but the colony displays signs that can be distinguished. These signs included White Syndrome, Skeletal Eroding Band, Brown Band, Black Band and other cyanobacterial infections [47], tissue necrosis caused by sediments containing a high mucus load and low numbers of cyanobacteria, algal interactions with corals that cause tissue erosion, bleached white patches, and pink coloration [48], [49]. Predator feeding scars from *Drupella* spp. and *Acanthaster planci* were identified and excluded from the analysis. Corals showing signs of compromised health were used to analyze the relationship between the number of unhealthy and healthy corals at each site. The prevalence of unhealthy corals was calculated by dividing the numbers of unhealthy corals by the total number of corals on each transect. Only scleractinian corals were used in the assessment.

### Percent cover of benthic organisms

Quantitative assessments of the benthos were made using the photoquadrat method [50]. Ten points were randomly selected and surveyed per transect (i.e., the same transects that were laid for the coral health surveys). At each point a photograph was taken using an Olympus 7070 digital camera that was connected to a tripod (1 m high) and a frame (0.9×0.6 m or 0.54 m^2^). Therefore, for each site 20 photoquadrats were sampled. During surveys, notes were made for each photoquadrat and collections were made for organisms that were unidentifiable *in situ*. Upon return to the research vessel, the twenty photographs were numbered and color adjusted using Adobe Photoshop v. 7.0. Image analysis was completed using the program Photogrid 1.0. For each photograph, 100 points were randomly generated and the organism under each point was identified. Therefore, for each site 2000 points were quantified. All organisms were identified to the finest level of resolution possible (genus level for scleractinian and soft corals, functional group for algal turfs and crustose coralline algae, and species level for macroalgae and macroinvertebrates where possible). The percent of coral cover was calculated by dividing the number of points that were assigned to scleractinian corals by the total number of points counted for each photoquadrat. Percent cover of scleractinian corals was used to identify the density dependent nature of the distribution of unhealthy corals on each atoll.

## Results

### Microbial and viral abundances

**Figure 2:**
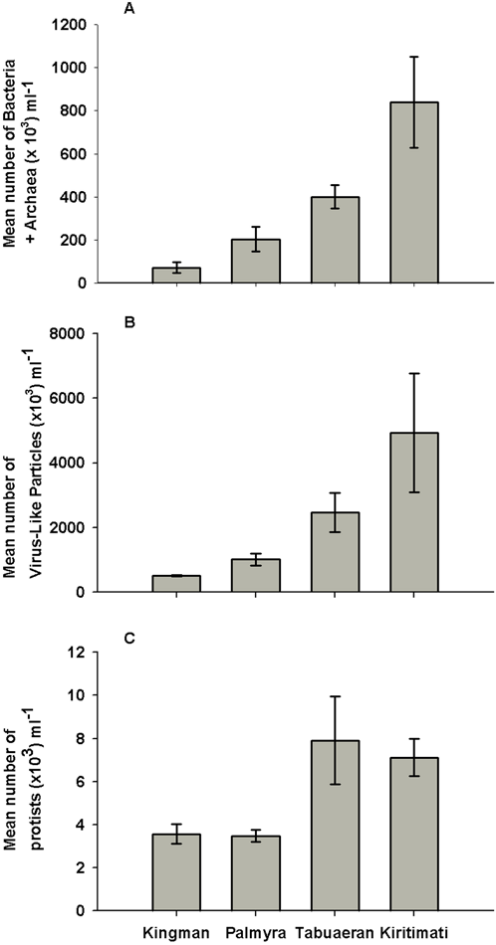
Direct counts were used to determine the mean abundance (±standard error) of A) microbial cells (Bacteria and Archaea), B) virus-like particles (VLPs), and C) protists on the four Northern Line Island atolls.

The microbial abundances (Bacteria and Archaea) and virus-like particles (VLPs) both increased by about an order of magnitude from Kingman to Kiritimati (Figure 2A and B; *F*_3, 12_ = 11.5, P<0.001 for microbes; *F*_3, 12_ = 10.5 P<0.001 for VLPs). In both cases, the increase was progressive from Kingman (7.2×10^4^±1.7×10^4^ microbes ml^−1^ and 5.1×10^5^±1.5×10^5^ VLPs ml^−1^) to Palmyra (2.0×10^5^±1.7×10^4^ microbes ml^−1 ^and 1.0×10^6^±3.7×10^5^ VLPs ml^−1^) to Tabuaeran (4.0×10^5^±2.3×10^4^ microbes ml^−1^ and 2.5×10^6^±1.2×10^6^ VLPs ml^−1^) to Kiritimati (8.4×10^5^±1.4×10^5^ microbes ml^−1^ and 4.9×10^6^±1.1×10^6^ VLPs ml^−1^) (Wilcoxon one–sided pairwise test was significant for all atoll pairings at the P = 0.05 level). Protist abundance doubled from Kingman and Palmyra (3575±457.1 and 3486±275.4 protists ml^−1^, respectively) to Tabuaeran and Kiritimati (7917±2037.1 and 7124±868.1 protists ml^−1^, respectively) (*F*_3, 12_ = 15.5, P<0.001; Figure 2C).

### Taxonomical and metabolic potential of microbial metagenomes

**Figure 3:**
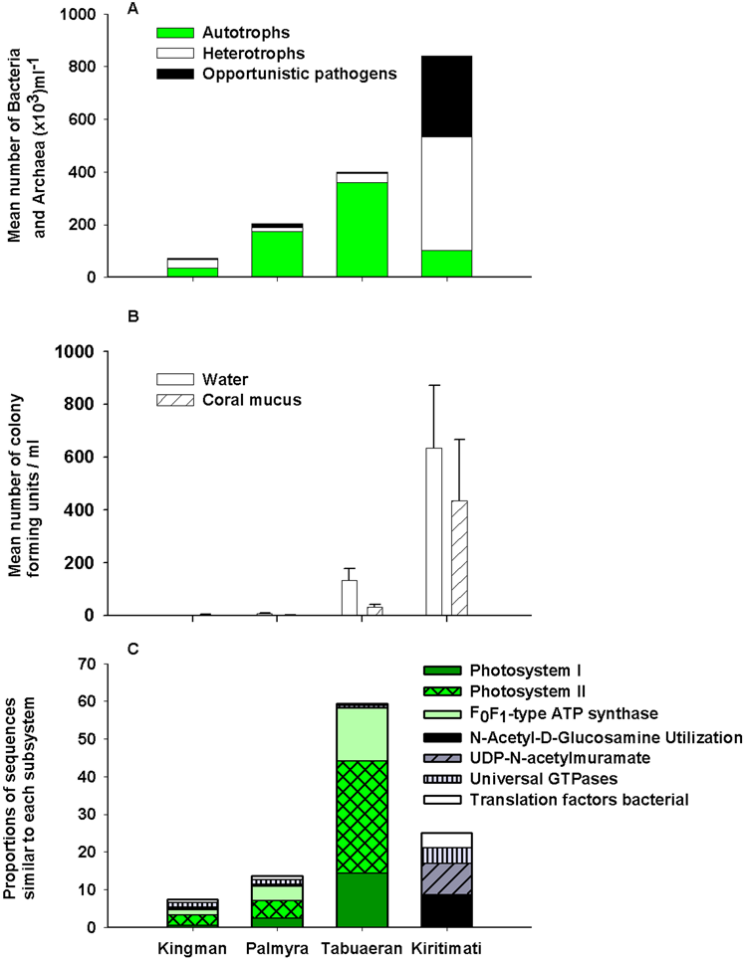
Taxonomic and metabolic potential of Bacteria and Archaea of the four atolls: A) Proportion of autotrophs, heterotrophs and potential pathogens identified by the 16S rDNA sequences in the microbial metagenomic fractions. B) Number of cultured *Vibrio* spp. (bar represents means±standard error) in the water column (F*_3,58_* = 5.697, P = 0.002, Wilcoxon one-sided paired t-test showed significant differences for all atoll pairings at P = 0.05) and coral mucus (F*_3,42_* = 3.514, P = 0.023, Wilcoxon one-sided paired t-test showed significant differences for all atoll pairings at P = 0.05, except between Kingman and Palmyra P = 0.299). C) The metabolic potential expressed by the seven most abundant subsystems, across the atolls. These subsystems varied significantly between Kingman and Kiritimati using both XIPE [37] and G-test (Supplementary data). Subsystems that are more closely associated with autotrophs are shown in green. The “potential pathogen” designation are known human pathogenic genera like *Staphylococcus*, *Vibrio*, and *Escherichia*, fish pathogens like *Aeromona*, and plant pathogens from the *Xylella* genera.

Taxonomic analyses of sequences from the large metagenomic fraction showed a non-monotonic change in the relative fractions of autotrophs and heterotrophs on the atolls (Figure 3A). Sequence comparisons with the 16S rDNA database showed that the microbial communities were increasingly autotrophic moving from Kingman (50% of identifiable metagenome sequence were similar to known autotrophs) to Palmyra (84%) to Tabuaeran (89%), but at Kiritimati the proportion of autotrophs sharply declined to 12% (Figure 3A). The robustness of this trend was supported further by comparisons of the DNA sequences against the SEED platform [36] and the Pfam database [43], which revealed similar changes in the relative proportion of autotrophs across the atolls (Figure S2). Further, the proportion of heterotrophs that were potential pathogens also increased on Kiritimati. The number of culturable *Vibrio* spp. from the water column and coral mucus samples also increased progressively from Kingman to Kiritimati (Figure 3B).

The metabolic potential of the microbial community, determined by comparing the sequences to the SEED platform and categorizing them into metabolic subsystems [36], showed similar patterns. Changes in relative abundance of autotrophic subsystems across atolls paralleled the non-monotonic changes described by the taxonomic analyses (Figure. 3C and S3). On Kingman, Palmyra, and Tabuaeran, sequences similar to the Photosystem I and II comprised 3.4, 7.2, and 44.3% of the total identifiable subsystems, respectively, but only 0.3% on Kiritimati (Figure 3C). F_0_F_1_-type ATP synthase, a subsystem that is involved in oxidative phosphorylation, showed a qualitatively similar change as the photosynthetic subsystems; F_0_F_1_-type ATP synthase is often coupled with photosynthesis to produce ATP. The N-Acetyl-D-glucosamine utilization subsystem, which is used in the consumption of fixed carbon and thus associated with heterotrophic growth, was highly represented on Kiritimati (8.2% of the identifiable sequences). In comparison, this subsystem was less than 1% on the other three atolls. Universal Guanosine Triphosphatase (UTPase), Uridine diphosphate-N-acetylmuramate and various translation factors were also highly represented on Kiritimati. Variation in less abundant metabolic subsystems across these atolls is provided in Figure S3.

The types of bacterial autotrophs in the microbial fraction also changed on the atolls. The most common bacterial autotrophic genus on Kingman and Palmyra was *Prochlorococcus* (75 and 91% of the cyanobacterial population, respectively), whereas on Tabuaeran and Kiritimati, *Synechococcus* was the most common genus (66 and 64% of the cyanobacterial population, respectively; Figure S4). This pattern may reflect variations observed in the water chemistry across the atolls, because *Prochlorococcus* is common in oligotrophic water, whereas *Synechococcus* becomes dominant in increasingly nutrient rich water [51], [52].

### Viromes

**Figure 4:**
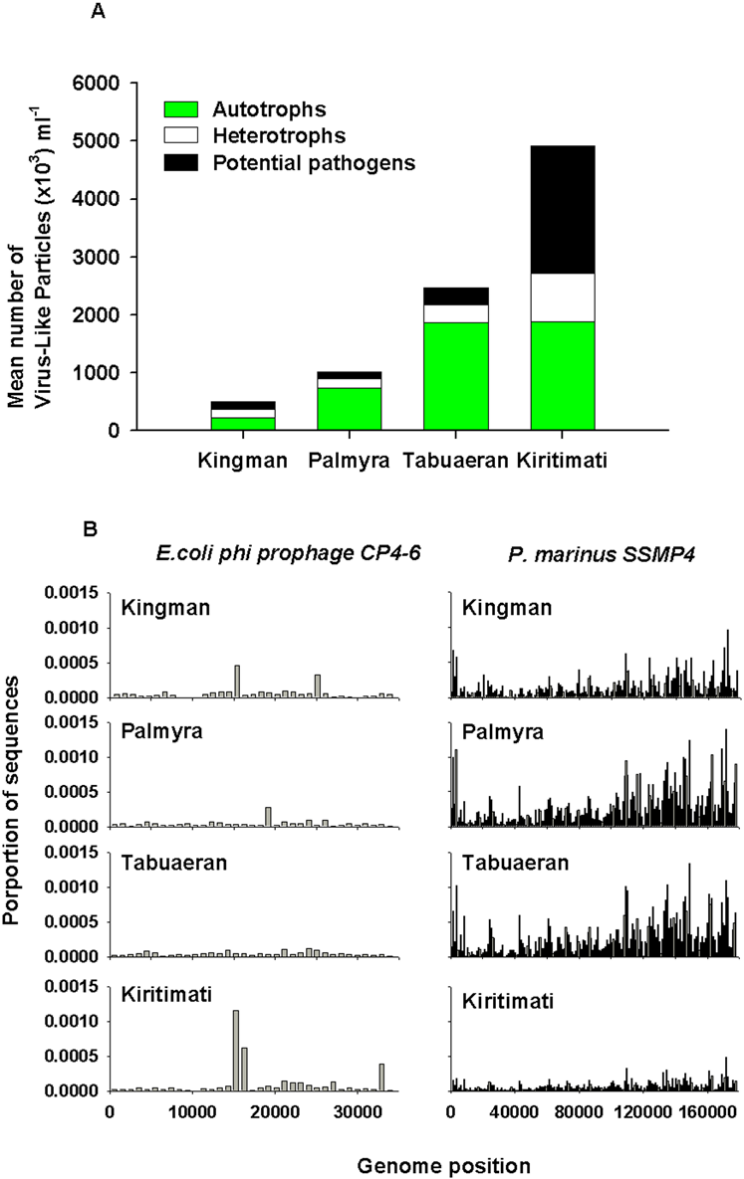
Analysis of the viral metagenomes showing: A) The relative abundances of phage host range by guild. This was the product of the mean number of virus-like particles and the proportion of sequences within the small metagenomic fraction that were similar to autotrophic, heterotrophic or potential pathogenic phage hosts. B) Sequence recruitment across the *Escherichia coli* Φ CP4-6 prophage (which is found in highly virulent *E. coli*) and *Prochlorococcus marinus* SSMP4 (which infects an open water autotrophic cyanobacteria).

The viral metagenomic fraction was compared to a database of all known phage and prophage genome sequences (http://phage.sdsu.edu/phage). Significant similarities to this database (E-value≤0.001) were used to identify the types of phages on each atoll. Since phage are host specific the proportion of phage infecting autotrophic and heterotrophic microbes was calculated. In parallel with the microbial analysis, the analysis of the phage hosts showed the phage known to infect cyanobacteria increased from Kingman (44%) to Palmyra (73%) and Tabuaeran (61%), and then at Kiritimati the phage known to infect heterotrophic microbes became dominant (61%; Figure 4A). A further breakdown of the potential host range of the phage is provided in the Figure S5.

The virome sequences were also analyzed using a fragment recruitment method to known genomes (described in [29]), which maps sequences to their relevant position on the reference genome (Figure 4B). Sequences similar to *Escherichia coli* Φ CP4-6 prophage, which is found in highly virulent enterohemorrhagic *Escherichia coli* strains [53], were more common on Kiritimati. In contrast, sequences similar to the *Prochlorococcus marinus* SSMP4 phage were more common in Kingman, Palmyra, and Tabuaeran (Figure 4B). The differences between the sequence distributions also became apparent when the average number of sequences showing similarities to each section of the genome was compared. For example, the number of sequences similar to *Escherichia coli* Φ CP4-6 prophage steadily increased from Kingman (29 sequences per 5000 bp), to Palmyra (66 sequences per 5000 bp) to Tabuaeran (91 sequences per 5000 bp) to Kiritimati (147 sequences per 5000 bp).

### Microbial predator-prey ratios

**Figure 5:**
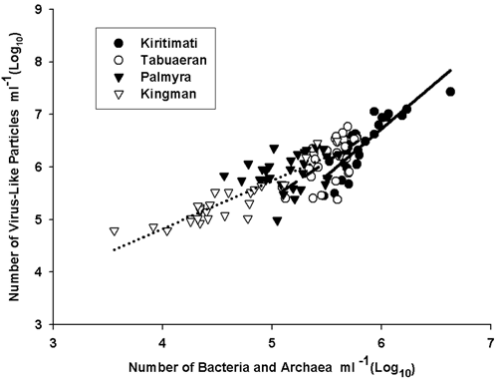
Relationships recorded between microbes and virus-like particle numbers on the Northern Line Islands. Kingman (dotted line) r^2^ = 0.807, P<0.0001; Palmyra r^2^ = 0.039, P = 0.414; Tabuaeran (dashed line) r^2^ = 0.324, P = 0.006; Kiritimati (solid line) r^2^ = 0.706, P<0.0001.

Virus-like particles (VLPs) and microbial numbers were positively correlated on Kingman, Tabuaeran, and Kiritimati, but not on Palmyra (Figure 5). The steepness of the slope of the VLPs:microbes increased from Kingman (0.909) to Tabuaeran (1.378) to Kiritimati (1.768). Microbes on Kiritimati were able to sustain approximately two times the number of VLPs than on Kingman, which suggests that the characteristics of the relationship are not static, but may be associated with conditions on each atoll.

The overall abundances of the protists increased from Kingman to Kiritimati, but the protists:microbe ratio declined. There were 0.0301 (±0.018) protists per microbial cell at Kingman, 0.013 (±0.005) at Palmyra, 0.015 (±0.004) at Tabuaeran, and 0.008 (±0.004) at Kiritimati. On Kingman, 66% of protists were strict heterotrophs (i.e., contained no chlorophyll) compared with 22% on Kiritimati.

### Coral cover and disease prevalence

**Figure 6:**
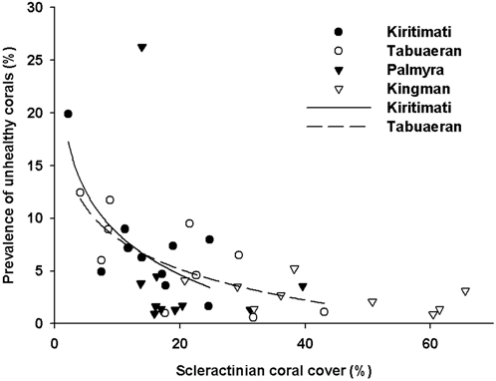
Prevalence of unhealthy scleractinian corals compared with scleractinian coral cover. The prevalence of unhealthy corals was negatively related to host density on both Tabuaeran (r^2^ = 0.477, P = 0.002) and Kiritimati (r^2^ = 0.664, P = 0.003). No relationship was found on Palmyra (r^2^ = 0.261, P = 0.141) or Kingman (r^2^ = 0.251, P = 0.300).

As shown in Figure 6, coral cover declined from Kingman (43.8%±5.4) to Palmyra (20.4%±2.3) to Tabuaeran (19.5%±1.0) to Kiritimati (14.9%±2.3), whereas prevalence of disease on hard corals was lowest on Kingman (2.5%±0.5) and highest on Kiritimati (6.3%±1.4) and Tabuaeran (6.2%±1.4). Palmyra showed medium prevalence of disease (4.8%±2.0) (Kruskal Wallis test; H = 8.0, df = 3, p = 0.04).

### Water chemistry

**Figure 7:**
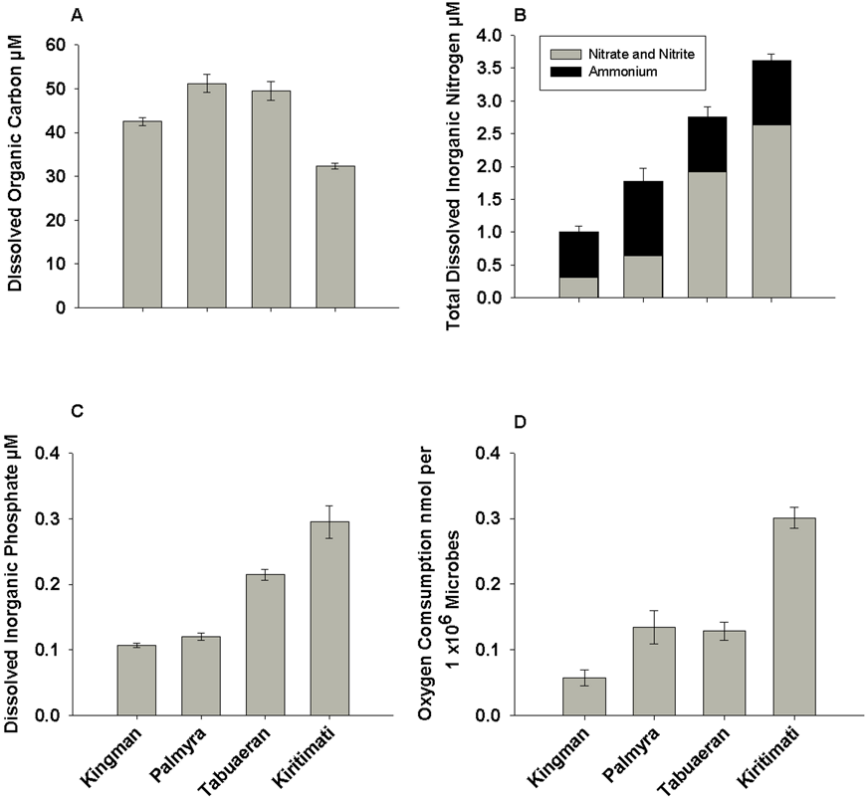
Water chemistry measured for the four Northern Line Island atolls. Concentrations of A) Dissolved organic carbon (DOC), B) Total dissolved inorganic nitrogen (TDIN: nitrite and nitrate, and ammonium), and C) Dissolved inorganic phosphate are presented as means (±standard errors). D) Microbial respiration rates as determined by adding the same microbial communities to samples of seawater collected from the four atolls.

Dissolved organic carbon (DOC) concentrations were highest on Palmyra (51.1±2.1 µmol l^−1^) and Tabuaeran (49.5±2.4 µmol l^−1^), lower on Kingman (42.5±0.9 µmol l^−1^), and lowest on Kiritimati (32.3±0.6 µmol l^−1^) (Figure 7A). Given the low numbers of measurements of DOC on coral reefs, a range of these values has been provided in Table S2 for comparison.

Total dissolved inorganic nitrogen (TDIN) increased almost four-fold from Kingman (1.3±0.08) to Kiritimati (3.6±0.1) (Figure 7B; F_3,12_ = 38.735, P<0.001), and similarly inorganic phosphate concentrations increased from Kingman (0.1±0.003) to Kiritimati (0.3±0.024) (Figure 7C; F_3,12_ = 395.2, P<0.001). No clear pattern was apparent in the concentrations of particulate organic carbon and particulate organic nitrogen (data not shown). No significant differences were found in the δ^15^N_Norm_ values in the particulate organic matter from Kingman (4.2±0.78) and Kiritimati (5.7±1.5) (not measured on Palmyra or Tabuaeran).

These differences in water chemistry were also reflected in results of the assays of oxygen consumption rates of a standard microbial community grown in seawater from each of the atolls. Microbes grown in water from Kingman had the lowest respiration rates (0.058±0.012 nmol oxygen consumed per 1×10^6^ microbes), whereas the same microbes grown in water from Kiritimati had much higher respiration rates (0.309±0.016 nmol oxygen consumed per 1×10^6^ microbes) (Figure 7D; P<0.001).

## Discussion

Microbial numbers in the water column overlying coral reefs usually range from 2–6×10^5^ cells ml^−1^ [2], [54], [55]. Our mean values were roughly comparable, although the lowest and highest mean values observed exceeded this range: Kingman averaged 7.2×10^4^ microbes ml^−1^, Palmyra averaged 2.0×10^5^ microbes ml^−1^, Tabuaeran averaged 4.0×10^5^microbes ml^−1^, and Kiritimati averaged 8.4×10^5^microbes ml^−1^. Reports for viral like particles (VLPs) range from 0.3–1.25×10^7^ VLPs ml^−1^ in the water column [56], densities that exceeded those were not observed: Kingman averaged 5.1×10^5^ VLPs ml^−1^, Palmyra averaged 1.0×10^6^ VLPs ml^−1^, Tabuaeran averaged 2.5×10^6^ VLPs ml^−1^, and Kiritimati averaged 4.9×10^6^ VLPs ml^−1^. For both microbes and VLPs, densities increased steadily across the four atolls; protists also increased, although in a stepwise fashion. There were also differences in community composition, most notably a sharp increase in heterotrophic Bacteria and Archaea and in potential pathogens in Kiritimati. Finally, we observed a steady increase in total dissolved inorganic nitrogen, which was 4-fold higher on Kiritimati than Kingman, and a similar pattern for inorganic phosphate, which increased 3-fold. In contrast, dissolved organic carbon (DOC) concentrations were highest on Palmyra and Tabuaeran and lowest on Kiritimati.

**Figure 8:**
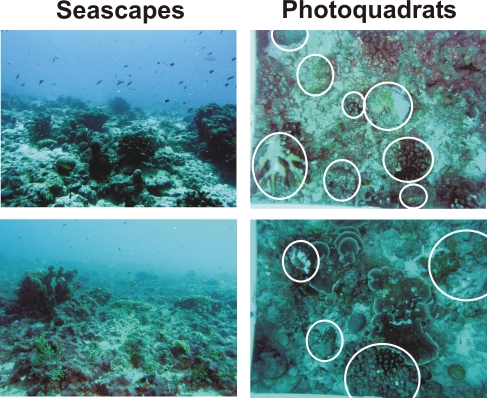
Seascape and photoquadrat photographs obtained from the metagenomic sampling site on Kiritimati. White circles indicate diseased, bleached, or recently dead corals.

A study of the macrobiota conducted simultaneously with our microbial study documented equally striking changes. Fish biomass dropped steadily from 527 to 132 g m^−1^ from Kingman to Kiritimati, primarily due to the loss of top predators. In parallel with these differences, coverage of corals and coralline algae declined from 71% to 21%, and cover by fleshy and turf algae increased from ∼20% to 68% from Kingman to Kiritimati [18]. For the macrobiota, historical data and data from nearby Pacific atolls [18] suggest that anthropogenic impacts are likely to be important factors in explaining these differences across the atolls. Historical records for Kiritimati indicate that sharks were once very abundant [57]–[60], and more recent surveys indicate a decline in fish biomass by 50% and coral cover by 30% in the last decade [61]–[63]; in contrast, uninhabited Kingman has not suffered such losses. The impact of people on fish communities is uncontroversial. The causes of coral loss are also likely to be anthropogenic, but the relative importance of local impacts (fishing and water quality) vs. global impacts (especially warming and associated bleaching) is more debated. Also Jarvis, an uninhabited island with similar oceanographic conditions and global warming conditions as Kiritimati, resembles Kingman in fish and benthic community structure [61]. One anecdotal consideration that suggests the decline of corals on Kiritimati appears to be a relatively recent event (i.e., within the last decade) is shown in seascape photos in Figure 8. Large coral skeletons were still free-standing in the algal-dominated reef areas and many of the still-living coral colonies were relatively large, with high levels of partial mortality (circles in photoquadrates). Similarly, surveys conducted in 1997 for a proposed Japanese space site identified higher coral cover on Kiritimati than was recorded in 2007, suggesting that the loss of corals is a recent event [63]. In sum, the historic data and the comparisons with nearby atolls suggest that the benthic and fish communities were originally similar on Kiritimati and Kingman in recent time [18].

However, for the microbes, there have been no systematic surveys on these atolls, including Jarvis, so interpreting the patterns observed is more complex. Microbial communities respond to the characteristics of seawater, which are affected by regional oceanographic differences, including local upwelling, lagoonal influences, land run-off, and the benthic community structure (especially the amount of benthic algae). The last three of these can be affected by both the physical and oceanographic characteristics of the atolls, by the activities of people locally, and anthropogenic global change. There are thus two competing, but not mutually exclusive, hypotheses to explain the observed microbial and macrobiota patterns in the Northern Line Islands (outlined in Table 1). Since the four atolls are different sizes and are separated by ∼750 kilometers along a north-south transect, regional differences and/or reef hydrology may be the primary driving factors for differences in the measured parameters. Alternatively, the varying levels of human disturbance associated with sewage (there is little industry or large-scale agriculture) and fishing that the atolls experience, or varying amounts of temperature stress associated with global warming may account for the observed differences.

**Table 1:** Summary of observations and possible interpretations of microbial and macro-organism data collected from the Northern Line Island survey.

Change as observer moves from Kingman to Kiritimati	Interpretation for hydrology/regional hypothesis	Interpretation for Human-driven food web shift hypothesis
Increased nutrients	a) Upwelling	a) Upwelling
	b) Terrestrial runoff (guano, sewage, agriculture, vegetation)	b) Terrestrial runoff
		c) Increase nitrogen fixation by cyanobacteria/turf algae*
Increased # of microbes and viruses	More microbes come from the larger lagoons	a) Overfishing of herbivores leads to more labile DOC
	Why do the herbivores not graze down the new algae?	b) Increased nutrients lead to more photosynthesis and DOC for microbes
Change from autotrophic to heterotrophic microbial communities	???	More labile DOC to support heterotrophs from unchecked macroalgae growth
More culturable *Vibrio* spp. and pathogen-like heterotrophs	???	Shift in types of *Vibrio* spp. due to DOC lability
*Prochlorococcus* to *Synechococcus* & autotrophic protists	Increased nutrients due to upwelling	Increased nutrients due to upwelling
Decreased coral cover	a) There were never corals in surveyed regions of Kiritimati	a) Overfishing increases labile DOC, increased coral-algal interaction zones, & pathogen reservoirs
	b) Coral bleaching killed corals	b) Coral bleaching leads to increased disease incidences
	Why do the Kiritimati corals look recently dead?	
Increased algal cover and shift from coralline to fleshy/turf algae	Nutrients favor fleshy and turf algae	a) Overfishing leads to decreased grazing
		b) Nutrients favor fleshy and turf algae
Increased coral disease	???	More pathogen-like microbes = more disease
Non-linear change in coral cover/disease prevalence	???	Same as above
Lower coral recruitment	Algae occupy substratum	a) Pathogens kill off recruits
		b) Algae occupy substratum
Losses of top predators in historical records	Bleaching destroys structure and fish leave	Overfishing
Inverted food pyramids for fish	Same as above	Same as above

Two hypotheses are considered: **Hydrology/regional hypothesis**-Larger islands are associated with more upwelling and algae. Different levels of bleaching on the various islands are the explanation of historical changes (i.e., loss of corals cause fish to leave). For example, the lagoons of Kiritimati and Tabuaeran may have served as sources of hot water during a warming period in the region. **Human-driven food web shift hypothesis**-Overfishing increases macroalgae, which increases amount of labile dissolved organic carbon (DOC). In turn the DOC increases heterotrophic/pathogenic microbes, which kill corals. These two hypotheses are not mutually exclusive. For example, algal-microbial dominated system may represent an alternate stable state initiated by a bleaching event. Where multiple interpretations are given, they are ranked in order of possible importance. Some questions for consideration are highlighted in red.

* Decreased grazing leads to higher concentrations of turf algae [18], [94]. These turfs contain cyanobacteria that fix nitrogen.

Some of the differences among the atolls are probably long-standing and reflect oceanographic and hydrographic differences, with predictable consequences for microbial community composition. Moving south from Kingman, the atolls are progressively larger with consequently greater potential to induce upwelling, larger lagoons, and larger seabird populations. All of these trends could influence microbial communities. For example, on Kingman, the autotrophic and heterotrophic microbial communities in the water column were roughly balanced. In oligotrophic waters, photosynthetic cyanobacteria are the major energy producers [64] and compete with the heterotrophic bacterial communities for inorganic nutrients [52]. *Prochlorococcus* utilizes reduced forms of nitrogen and loses competitive dominance in seawater where the levels of nitrates are high [64], [65]. On Tabuaeran, the photosynthetic microbes made up 80% of the community and photosynthetic subsystems comprised over 40% of the sequences identified within the metagenome. The dominance by *Synechococcus* correlated with the increase in nitrogen and phosphate concentrations in the water and is similar to the large scale distribution patterns of autotrophs in the ocean [64]. The increase in photosynthesis on Palmyra and Tabuaeran may have caused the increased concentration of DOC on these atolls (Figure 7A). Similarly, the metagenomes showed that the number of microbial autotrophs in the 0.45–100 micron fraction increased from Kingman (50%) to Palmyra (84%) to Tabuaeran (89%) (Figs. 3A and C). This trend correlated well with the increasing concentration of fixed nitrogen compounds (nitrate, nitrite, and ammonium) and phosphate in the water column (Figures. 7B and C) and may be due to increased upwelling on the progressively larger atolls. The concentrations of nitrate/nitrite and phosphate continue to increase on Kiritimati, but the microbial community became predominantly heterotrophic in nature (72%), suggesting an available carbon source. This observation is consistent with the hypothesis that nutrients from upwelling, and possibly runoff from the island, combined with a loss of herbivory are stimulating benthic macroalgae and phytoplankton. In turn, the algae produces dissolved organic carbon (DOC) which supports more heterotrophic bacterial growth. This is additionally supported by the observation that both the algal cover and the highest numbers of microbes were also observed on Kiritimati.

The apparent inconsistency with the hypothesis that high levels of DOC released by algae are increasing heterotrophic bacterial is that the lowest DOC concentrations were observed on Kiritimati. Similar phenomena have been observed on other coral reefs in the Caribbean and Sri Lanka (strong correlation between higher microbial numbers and lower DOC; Pantos, Fairoz, Rohwer; unpublished data). While this may seem counter-intuitive, the lower DOC concentrations are the result of co-metabolism of refractory carbon sources that occurs when microbes are given an excess labile carbon. Carlson et al. [66] showed that increases in inorganic nutrients alone were insufficient to enable bacterial communities to utilize refractory DOC, but required an addition of a bio-available source of DOC. Further, when the labile carbon was supplied, the taxonomic composition of the microbial communities changed (the study by Carlson et al, however, did not identify the microbes) [66], similar to the differences in taxonomic composition that were observed across the four coral atolls. Similarly, fresh carbon supplied to soil microbes enabled the mineralization of old carbon [67]. Consistent with this explanation, addition of the same laboratory microbial community to seawater samples from all four coral atolls showed that the lower DOC-containing water from Kiritimati supported more microbial respiration (Figure 7D).

Total nitrogen inputs associated with sewage were estimated to be 227 and 397 kg N^−1^ km^−1^ yr^−1^ for the inhabited coastline of Tabuaeran and Kiritimati, respectively [18]. Given the large volumes of water that moves passed these reefs, we expect that this extra nitrogen from sewage will be diluted out. While these nutrients may have influenced the microbial community to some extent, they are a fraction of the inputs estimated on highly populated reefs, such as Florida Bay [68]. Additionally, no evidence of human sewage was apparent in the isotopic signature of the particulate organic matter δ^15^N_Norm_ values from Kiritimati (5.7±1.5) compared with Kingman values (4.2±0.78). Therefore, human-derived sewage does not seem to be the reason for the elevated nutrients on Tabuaeran or Kiritimati. Bird guano, however, is a potential influence that was not controlled for in this study and may explain some of the elevated nutrient concentrations on Kiritimati, Tabuaeran, and Palmyra.

Increasing atoll size and oceanographically more oligotrophic water were directly correlated with significant increases in protists, microbes, and VLPs. However, the decreasing percentage of heterotrophs from Kingman to Palmyra, followed by an abrupt shift to a heterotroph dominated-community on Kiritimati, does not directly match this pattern. The most straight-forward explanation, as presented above, is that an increase macroalgae, and possibly phytoplankton, is producing labile DOC that supports the change in the microbial community on Kiritimati.

Disease incidence on coral reefs are associated with human activities [69], [70]. Changes in the chemical composition of seawater may affect coral disease levels, either by favoring the growth of pathogens and/or decreasing the resistance of the coral animal to infection. Increases in inorganic nutrients are typical on coral reefs influenced by human activities and have been implicated in increasing severity of fungal infections of corals [71]. However, recent experiments suggest that dissolved organic carbon (DOC) may also be important. Experimental dosing of coral fragments with increased inorganic nutrients did not increase coral mortality, but the addition of DOC caused tissue necrosis and mortality [72], [73] and increased microbial growth. Another common coral stressor, sedimentation, also causes coral tissue loss and mortality in the presence of high organic material [74], [75]. Treatment of organic laden sediments with antibiotic stopped the coral mortality [76]. Smith et al. [77] showed that corals died when placed adjacent to macroalgae, even when separated by a 0.02 µm membrane that was impermeable to viruses and microbes, but not dissolved compounds like DOC. The algae increased microbial growth on the coral, which in turn caused hypoxia and presumably the coral mortality. Coral mortality did not occur in this experiment when antibiotics were added [77]. These results suggest that algal-derived DOC may be a primary driver of coral-microbial interactions. In addition, algae-associated microbial communities harbor pathogens that cause coral disease [78].

Potential pathogens were proportionately more abundant in the Kiritimati microbial metagenomic sample (36.3%; Figure 3A), including many bacterial genera and species that are known pathogens of eukaryotes (Figures. 3A 3C and S5) and human pathogens like *Staphylococcus, Vibrio,* and *Escherichia.* The culturable *Vibrio* spp. data support this observation (Figure 3B), as do the metagenomic analyses of the viromes (Figures 4 and S5). While it is not possible to absolutely prove (because of microbial genomic plasticity) that these cultured and uncultured data represent pathogens, the combined data is indicative of unhealthy waters. The increase in potential pathogens could be caused by changes in DOC, which stimulates heterotrophic microbial growth or by increased input of pathogens from the humans and animals living on Kiritimati. The human introduction of pathogens suggested for *Serratia* spp. infection of acroporid corals in the Florida Keys [79], but may be less likely on Kiritimati given the lack of sewage signature.

Whatever the source, increases in potential pathogens may contribute to the documented recent loss of corals and present patterns of prevalence of disease. Doubling the concentration of culturable *Vibrio* spp. or enteric-like microbes in the water column caused 100% coral mortality under experimental conditions [72]. Therefore, the observed ten-fold increase in abundance of microbes, in both the direct counts and by culturing, has the very real potential of killing corals in Kiritimati.

The hypothesis that the Kiritimati microbial community is detrimental to corals raises the important question: Is this type of microbial community something that should be expected on coral reefs? The sampling scheme used in this study did not find regions of high heterotrophic activity on Kingman, Palmyra, or Tabuaeran. The sampling was performed at defined distance intervals, which resulted in a more complete survey of the smaller islands. However, a possibility remains that we failed to find the right area on the other atolls that had the higher microbial communities. Regional differences are also a possible explanation for the observed data. Kiritimati may have bleached in the relatively recent pass (a good candidate is the 1998 warming event) [18]. If this event killed the corals, then algae could have colonized the area. In this case the microbial mechanisms discussed above could help prevent recolonization by corals.

The hypothesis we favor, however, is that a change in the food web structure explains the observed differences. On Kingman and Palmyra, there was no significant relationship between disease prevalence and host density, whereas disease prevalence was negatively related to host density on Tabuaeran and Kiritimati (Figure 6). Generally, a density dependent relationship exists between the hosts and pathogens, with the prevalence of disease increasing with host density [80]. The loss of the density dependent nature of the host-pathogen relationship suggests environmental factors are increasing opportunistic coral diseases. The proposed mechanism is that overfishing removes both predatory and herbivorous fish. Loss of the herbivorous fish results in more algae and microbial growth, which leads to an increased coral death via the microbial mechanisms described above. Removal of the top predators (i.e., top down control) slows down the rate at which energy turns over in the system. This extra energy, in the form of DOC, supports more heterotrophic microbes. Obviously, this is a complex feedback between fish, algae, microbes and coral health that requires further investigation.

### Future studies to differentiate between regional/hydrological and food web hypotheses

Table 1 outlines a number of observations and their interpretation in the context of the two competing hypotheses. The main differences revolve around the ultimate cause of coral reef decline. Global and regional phenomena are the major factors structuring coral reefs and their geotemporal rise and decline. The current global decline in coral reefs, however, is almost certainly human-driven. Coral bleaching, caused by rising sea surface temperatures, can devastate coral reefs. Microbes are assuredly important components of this stress, either as primary causes [81]–[84] or as opportunistic pathogens that kill the weakened corals. Bleaching and other perturbations that destroy the structure of the reef appear to drive coral reefs into another stable state and yield observations similar to what was observed on Kiritimati. Cores will be able to determine if the areas outside of the lagoon have always had low coral cover, or if this is a relatively recent event as suggested by Figure 8. A complete survey of Kiritimati will be able to determine if the rest of the atoll (which includes areas that are not fished or adjacent to villages) has lost its coral cover and subsequent fish populations. If the coral communities are still in place, this would argue against a large scale bleaching event as the triggering event. One caveat is that local hydrology could protect one part of the island, while another area bleaches. Again, cores should help differentiate between these possibilities. Surveys of additional coral reefs would help establish whether there are correlations between coral condition and changes in the microbial communities. The most straight-forward study to test the hypothesis that microbial numbers are driven by increased macroalgae growth and release of DOC, would be a caging experiment where grazers are added back to a degraded reef to determine if the microbial communities respond. While many caging experiments have been conducted (normally excluding herbivores), none have measured DOC and microbial numbers. Obviously understanding coral reef decline is an active area of research, and the survey presented here provides some insights into microbial involvement in that process. It is important to establish the mechanism driving changes in microbial growth and coral condition because of their importance for management actions.

### Conclusions

In the last thirty years, coral reefs worldwide have suffered an unprecedented loss of coral cover [85]. The positive correlation between human-associated disturbance and coral reef decline is now clear, but there is considerable debate about the precise mechanisms of coral loss. Research to identify these mechanisms has focused on the effects of overfishing, habitat destruction, tourism, global warming, and increases in nutrients from terrestrial run-off [86]–[88]. With the exception of direct destruction (cyanide, blasting, construction), it is not clear why corals actually die. Bleaching, while important does not always lead to coral mortality [89], direct overgrowth by algae is insufficient to explain the widespread loss of corals. An obvious common denominator in the major scenarios of coral death is disease caused by microbes, either as epidemics causes by specific microbes, such as white band disease which devastated acroporid corals in the Caribbean [90] or opportunistic pathogens as suggested on Kiritimati and Tabuaeran. Specific pathogens can also cause food web to shifts, such as the phase shift triggered by the disease of the sea urchin *Diadema* spp. in the Caribbean [91]–[93]. As in the overfishing food web shift proposed above, opportunistic pathogens were probably the ultimate cause of coral death after the sea urchin die-off. Ecosystem-based management of coral reefs has traditionally focused on animals and plants. Our findings highlight the need to explicitly include microbial processes and their influence on coral reef ecosystem function. Such a framework is also needed to elucidate factors that sustain coral health.

## Supporting Information

Figure S1 Underwater sampling equipment used to obtain the 150-liter water sample for the metagenomic analysis. The water was taken from the surfaces and crevices of the reef structure.
(1.01 MB TIF)

Figure S2 The taxonomic components of the large metagenomic fraction analyzed via sequence similarities to the A) whole genome within the SEED platform, and B) Pfam database.
(2.18 MB TIF)

Figure S3 The subsystems that showed differences between Kingman and Kiritimati.
(2.18 MB TIF)

Figure S4 Proportions of Prochlorococcus and Synechococcus present in the large metagenomic fraction.
(2.11 MB TIF)

Figure S5 The percentage of the predicted host range of phage in the small metagenomic libraries.
(2.18 MB TIF)

Table S1 Total number of sequences retrieved in each metagenomic library and the number that showed similarities to those stored in the SEED platform.
(0.03 MB DOC)

Table S2 Nutrient and organic carbon concentrations measured on coral reefs.
(0.10 MB DOC)
