# Supplementary figures and images for: Microbial Ecology of Four Coral Atolls in the Northern Line Islands
Source: PLoS One. 2008 Feb 27;3(2):e1584. doi: 10.1371/journal.pone.0001584 (PMC2253183; doi:10.1371/journal.pone.0001584)

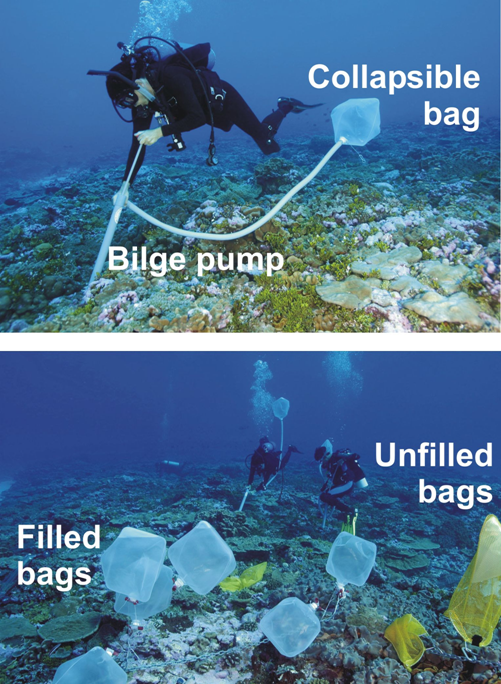

Supplement: Figure S1 — Underwater sampling equipment used to obtain the 150-liter water sample for the metagenomic analysis. The water was taken from the surfaces and crevices of the reef structure. (1.01 MB TIF) [file pone.0001584.s001.tif]
