# Supplementary material for: Microbial Ecology of Four Coral Atolls in the Northern Line Islands
Source: PLoS One. 2008 Feb 27;3(2):e1584. doi: 10.1371/journal.pone.0001584 (PMC2253183; doi:10.1371/journal.pone.0001584)
Supplement: Table S1 — Total number of sequences retrieved in each metagenomic library and the number that showed similarities to those stored in the SEED platform. (0.03 MB DOC) [file pone.0001584.s006.doc]

### **Supplementary Table T1.** Total number of sequences retrieved in each metagenomic library and the number that showed similarities to those stored in the SEED platform.

| **Sample** | **Number of Sequences** | **Similarities in SEED** | **Percent similarity** |
| --- | --- | --- | --- |
| Kingman Large Fraction | 188,445 | 22,130 | 11.74 |
| Kingman Small Fraction | 94,915 | 8,082 | 8.515 |
| Palmyra Large Fraction | 289,723 | 39,143 | 13.51 |
| Palmyra Small Fraction | 320,397 | 16,094 | 5.02 |
| Tabuaeran Large Fraction | 290,844 | 19,369 | 6.66 |
| Tabuaeran Small Fraction | 380,355 | 18,752 | 4.93 |
| Kiritimati Large Fraction | 227,542 | 37,604 | 16.53 |
| Kiritimati Small Fraction | 283,390 | 82,400 | 29.08 |
| **Total** | 2,075,611 | 243,574 | 11.74 |
