# Supplementary material for: Microbial Ecology of Four Coral Atolls in the Northern Line Islands
Source: PLoS One. 2008 Feb 27;3(2):e1584. doi: 10.1371/journal.pone.0001584 (PMC2253183; doi:10.1371/journal.pone.0001584)
Supplement: Table S2 — Nutrient and organic carbon concentrations measured on coral reefs. (0.10 MB DOC) [file pone.0001584.s007.doc]

Supplementary Table S2. Nutrient and organic carbon concentrations measured on coral reefs.

| **Study** | **Location - Description** | **Nitrate**  **(μM)** | **Ammonia**  **(μM)** | **Phosphate**  **(μM)** | **Organic Carbon**  **(μM)** |
| --- | --- | --- | --- | --- | --- |
| Cost et al 2000 [1] | Guarajuba Reef, Bahia Brazil  - polluted - | 2.3 - 12.1 | 4.8 – 10.7 | 0.1 - 1.6 | ND |
| Costa et al. 2006 [2] | Coroa Vermelha, Ponta Grande, Recife de Fora, Brizil | (TON)0.41-2.56 |  | 0.20-0.91 | ND |
| Hata et al., 2002 [3] | Shiraho reef, Ishigaki Is., Japan | ND | ND | ND | 2 – 4.5 (POC)  60 – 87 (DOC/HTCO) |
| Boyer, Fourqurean, &  Jones  1999 [4] | Florida Keys National Marine Sanctuary (154 stations) | 0.7 - 4.5 | 1.7 - 10.3 | 0.3 - 0.8 | 333 –1653 |
| Furnas et al., 1990 [5] | GBR multiple inner reef lagoons | o.65(se0.22) | 0.14 (se0.06) | 0.20 (se<0.01) | ND |
| Hatcher and Firth 1985 [6] | One tree Island, GBR reef slope | 0.31 (se0.03) | 0.76 (se0.24) | ND | ND |
|  | Lagoon | 0.95 (se0.06) | 2.86 (se0.21) | ND | ND |
|  | Reef crest tidal pools | 1.54 (se0.16) | 5.52 (se0.62) | ND | ND |
| Littler et al 1991 [7] | Seychelles Granite island | 0.5 | 1.94 | 0.12 | ND |
|  | Carbonate Island | 0.45 | 0.2 | 0.04 | ND |
|  | Carbonate Island with birds | 3.58 | 1.9 | 0.5 | ND |
| Koop et al. 2001 [8]  ENCORE | GBR - 2 year  nutrient enrichment | ND | Plus  11.5 – 36.2  (ammonium chloride) | Plus  2.3 – 5.1  (potassium phosphate) | ND |
| Lapointe 1997 [9] | Jamaica Bay | 4.96 – 27.9 | 0.19 – 0.49 | 0.10 – 0.33 | ND |
|  | Florida | 0.35 – 1.22 | 0.20 – 2.42 | 0.13 – 0.32 | ND |
| Marsh 1977 [10] | Tumon Bay, Guam  fringing reef w/ run off | 4.1 - 8.0 | ND | 0.1 -0.6 | ND |
| McCook 2001 [11] | Fringing reef @ Gould Island, GBR | ND | 0.1 - 0.2 | 0.1 - 0.2 | ND |
| Mallela and Perry 2007 [12] | Rio Bueno  Jamaica Low terrestrial run-off | 1.11-40.48 | 0.13-0.5 | 0.0-0.21 | ND |
|  | Medium terrestrial run-off | 0.39-39.77 | 0.18-1.10 | 0.02-0.23 | ND |
| Pages et al., 1997 [13] | Reef lagoon, Tuamotu Archipelago | ND | ND | ND | 58-167 (DOC/HTCO) |
| Torreton et al 1997 [14] | Tikehau lagoon, French Polynesia | ND | ND | ND | 105± 17.0 (SD)  (DOC/HTCO) |
|  | Great Astrolabe Reef Lagoon, Fiji | ND | ND | ND | 114 ± 7.0 (SD) (DOC/HTCO) |
| van Duyl and Gast 2001 [15] | Curacao Reefs, Netherland Antilles | ND | ND | ND | 154-208 (DOC/WCO) |
| van Duyl and Gast 2002 [16] | Curacao Reefs, Netherland Antilles | 0.5 – 1.5 | ND | 0.04 - 0.1 | 166 – 290 |
| Webber and Roff 1996 [17] | Offshore Discovery Bay,  Jamaica | ND | ND | ND | 6 – 66 |
| Yoshinaga et al 1991 [18] | Majuro Atoll Lagoon | ND | ND | ND | 145 ± 45 |
|  | Ponape Island Lagoon | ND | ND | ND | 223 ±89 |
| Yahel et al., 2003 [19] | Eilat, Israel | ND | ND | ND | 79-81 |
| This study | Kingman | 0.316±0.035 | 0.685±0.082 | 0.107 ± 0.007 | 42.5 ± 0.9 |
| This study | Palmyra | 0.638±0.080 | 1.133±0.157 | 0.120 ± 0.012 | 51.1 ± 2.1. |
| This study | Tabuaeran | 1.916±0.124 | 0.840±0.096 | 0.197 ± 0.016 | 49.0 ± 2.4 |
| This study | Kiritimati | 2.637±0.103 | 0.971±0.066 | 0.295 ± 0.036 | 32.3 ± 0.6 |

Organic carbon levels are reported as dissolved organic carbon (DOC; passes through a GF/F filter) or particulate organic carbon (POC). The two major ways that organic carbon is measured are high temperature combustion oxidation (HTCO) and wet chemical oxidation (WCO). In general, WCO is not considered reliable. Polluted is based on relevant authors’ notes. GBR = Great Barrier Reef; Queensland, Australia. This table is not meant to be exhaustive, but focuses on the reports of Dissolved Organic Carbon levels and demonstrates that very few assessments measure both inorganic and organic nutrients. Levels are reported as means and either standard error (se) or standard deviation (sd), or as a range, depending on the publication. TON is Total organic nitrogen, the addition of all dissolved nitrogen categories.

References

1. Costa O, Leão Z, Nimmo M, Attril M (2000) Nutrification impacts on coral reefs from northern Bahia, Brazil. Hydrobiologia 440.

2. Costa OS, Attrill MJ, Nimmo M (2006) Seasonal and spatial controls on the delivery of excess nutrients to nearshore and offshore coral reefs of Brazil. J Mar Sys 60: 63-74.

3. Hata H, Kudo S, Yamano H, Kurano N, Kayanne H (2002) Organic carbon flux in Shiraho coral reef (Ishigaki Island, Japan). MEPS 232: 129-140.

4. Boyer JN, Fourqurean JW, Jones RD (1999) Seasonal and long-term trends in the water quality of Florida Bay (1989-1997). Estuaries 22: 417-430.

5. Furnas M, Mitchell A, Gilmartin M, Revelante N (1990) Phytoplankton biomass and primary production in semi-enclosed reef lagoons of the central Great Barrier Reel Australia. Coral Reefs 9: 1-10.

6. Hatcher A, Frith C (1985) The control of nitrate and ammonium concentrations in a coral reef lagoon. Coral Reefs 4: 101-110.

7. Littler M, Littler D, Titlyanov E (1991) Comparisons of N- and P-limited productivity between high granitic islands versus low carbonate atolls in the Seychelles Archipelago: a test of the relative-dominance paradigm. Coral Reefs 10.

8. Koop K, Booth D, Broadbent A, Brodie J, Bucher D, et al. (2001) ENCORE: The effect of nutrient enrichment on coral reefs. Synthesis of results and conclusions. Mar Poll Bull 42: 91-120.

9. Lapointe BE (1997) Nutrient thresholds for bottom-up control of macroalgal blooms on coral reefs in Jamaica and southeast Florida. Limnol Oceanogr 42: 1119-1131.

10. Marsh JA. Terrestrial inputs of nitrogen and phosphorus on fringing reefs of Guam; 1977. pp. 331-336.

11. McCook LJ, Jompa J, Diaz-Pulido G (2001) Competition between corals and algae on coral reefs: a review of evidence and mechanisms. Coral Reefs 19: 400-417.

12. Mallela J, Perry CT (2007) Calcium carbonate budgets for two coral reefs affected by different terrestrial runoff regimes, Rio Bueno, Jamaica. Coral Reefs 26: 129-145.

13. Pages J, Torreton J, Sempere R (1997) Dissolved organic carbon in coral-reef lagoons by high temperature catalytic oxidation and UV spectrometry. C R Acad Sci Ser II A Sci Terre Planetes 324: 915-922.

14. Torreton JP, Dufour P (1996) Temporal and spatial stability of bacterioplankton biomass and productivity in an atoll lagoon. Aquat Microbial Ecol 11: 251-261.

15. van Duyl FC, Gast GJ (2001) Linkage of small-scale spatial variations in DOC, inorganic nutrients and bacterioplankton growth with different coral reef water types. Aquat Microbial Ecol 24: 17-26.

16. van Duyl FC, Gast GJ, Steinhoff W, Kloff S, Veldhuis MJW, et al. (2002) Factors influencing the short-term variation in phytoplankton composition and biomass in coral reef waters. Coral Reefs 21: 293-306.

17. Webber MK, Roff JC (1995) Annual Structure of the Copepod Community and Its Associated Pelagic Environment Off Discovery-Bay, Jamaica. Mar Biol 123: 467-479.

18. Yoshinaga I, Fukami K, Ishida Y (1991) Comparison of DNA and Protein-Synthesis Rates of Bacterial Assemblages between Coral-Reef Waters and Pelagic Waters in Tropical Ocean. MEPS 76: 167-174.

19. Yahel G, Sharp JH, Marie D, Hase C, Genin A (2003) In situ feeding and element removal in the symbiont-bearing sponge Theonella swinhoei: Bulk DOC is the major source for carbon. Limnol Oceanogr 48: 141-149.
